# Causal relationships between body mass index, low-density lipoprotein and bone mineral density: Univariable and multivariable Mendelian randomization

**DOI:** 10.1371/journal.pone.0298610

**Published:** 2024-06-13

**Authors:** Yuxiang Wu, Weiwei Ma, Zhenda Cheng, Qiwei Zhang, Zhaodong Li, Punan Weng, Bushuang Li, Zhiqiang Huang, Changlong Fu

**Affiliations:** 1 Quanzhou Hospital of Traditional Chinese Medicine, Quanzhou, Fujian, China; 2 Fujian University of Traditional Chinese Medicine, Fuzhou, Fujian, China; 3 School of Acupuncture-Moxibustion and Orthopaedics College of Acupuncture, Hubei University of Chinese Medicine, Wuhan, Hubei, China; 4 Department of Orthopaedics, Dongzhimen Hospital, Beijing University of Chinese Medicine, National Center for Traditional Chinese Medicine, Beijing, China; 5 Guangzhou University of Traditional Chinese Medicine, Guangzhou, Guangdong, China; 6 Department of body conditioning, Xiamen Hospital of Beijing University of Chinese Medicine, Xiamen, Fujian, China; 7 Academy of Integrative Medicine, Fujian University of Traditional Chinese Medicine, Fuzhou, Fujian, China; 8 Fujian Provincial Key Laboratory of Integrative Medicine on Geriatrics, Fuzhou, Fujian, China; Far Eastern Memorial Hospital, TAIWAN

## Abstract

**Summary:**

Utilizing the Mendelian randomization technique, this research clarifies the putative causal relationship between body mass index (BMI) andbone mineral density (BMD), and the mediating role of low-density lipoprotein (LDL). The implications of these findings present promising opportunities for enhancing our understanding of complex bone-related characteristics and disorders, offering potential directions for treatment and intervention.

**Objective:**

The objective of this study is to examine the correlation between BMI and BMD, while exploring the intermediary role of LDL in mediating the causal impact of BMI on BMD outcomes via Mendelian randomization.

**Methods:**

In this study, we employed genome-wide association study (GWAS) data on BMI, LDL, and BMD to conduct a comparative analysis using both univariate and multivariate Mendelian randomization.

**Results:**

Our study employed a two-sample Mendelian randomization design. Considering BMI as the exposure and BMD as the outcome, our results suggest that BMI may function as a potential protective factor for BMD (β = 0.05, 95% CI 1.01 to 1.09, *P* = 0.01). However, when treating LDL as the exposure and BMD as the outcome, our findings indicate LDL as a risk factor for BMD (β = -0.04, 95% CI 0.92 to 0.99, *P* = 0.04). In our multivariate Mendelian randomization (MVMR) model, the combined influence of BMI and LDL was used as the exposure for BMD outcomes. The analysis pointed towards a substantial protective effect of LDL on BMD (β = 0.08, 95% CI 0.85 to 0.97, *P* = 0.006). In the analysis of mediation effects, LDL was found to mediate the relationship between BMI and BMD, and the effect was calculated at (β = 0.05, 95% CI 1.052 to 1.048, *P* = 0.04).

**Conclusion:**

Our findings suggest that BMI may be considered a protective factor for BMD, while LDL may act as a risk factor. Moreover, LDL appears to play a mediatory role in the causal influence of BMI on BMD.

## 1. Introduction

Osteoporosis (OP) is a systemic metabolic bone disorder, typified by decreased bone mass, gradual loss of bone trabeculae, and reduction in bone mineral density [1]. Epidemiological studies have revealed that over 200 million individuals globally are afflicted with OP, with a prevalence of 19.20% among individuals over 50 in China [2]. It is projected that this figure will escalate in the future due to an aging global population [3]. Concurrently, owing to shifts in lifestyle patterns, obesity has manifested as a global epidemic trending towards a younger demographic, significantly impairing people’s health and quality of life. A study indicated that China’s mean BMI increased to 24.4 kg/m^2^ in 2018 from 22.7 kg/m^2^ in 2004, and the prevalence of obesity attained 8.1% [4]. Recent studies have unearthed a correlation between adiposity and osteoporosis. Conventionally, a relatively elevated body mass index (BMI) is considered clinically protective for human bones, and BMI also serves as an indicator for the fracture risk assessment tool (FRAX) [5]. However, this hypothesis has faced challenges as research advances, revealing that obesity has a detrimental impact on bone health. A contradictory outcome emerged from a 5-year clinical study conducted in Spain. In this study involving 250 postmenopausal women tracked over 5 years, obese postmenopausal women exhibited lower bone formation markers, while older obese women displayed higher bone resorption markers. This suggests a substantially elevated risk of osteoporosis in the obese population compared to the normal population [6].

The conflicting nature of these results arises from the myriad factors influencing adiposity and bone structure. Clinical studies are susceptible to various biases, confounding factors, and causal time constraints. These elements collectively contribute to the persistence of the "obesity paradox" in metabolic research [7, 8].Mendelian randomization analysis (MR), a method extensively utilized in clinical epidemiological research, leverages genetic diversity in genes as an instrumental variable to scrutinize causative correlations between exposure factors and outcome events [9]. MR investigations satisfy causal temporal order, underpinning causal linkages in causal inference [10]. Given genetic variants are determined at conception, they are generally less vulnerable to external influences or confounding. MR has been used to analyze the causal impact of obesity on circulating lipoproteins and the influence of lipids on BMD [11, 12]. While these studies may explain some of the protective mechanisms of BMI on BMD, they do not autonomously quantify mediating effects. This research, intending to investigate the relationship between BMI and BMD and the mediating role of LDL, has employed publicly available genome-wide association study data as the basis for two-sample MR and MVMR analyses.

## 2. Research methods and materials

### 2.1 Experimental design

**Fig 1:**
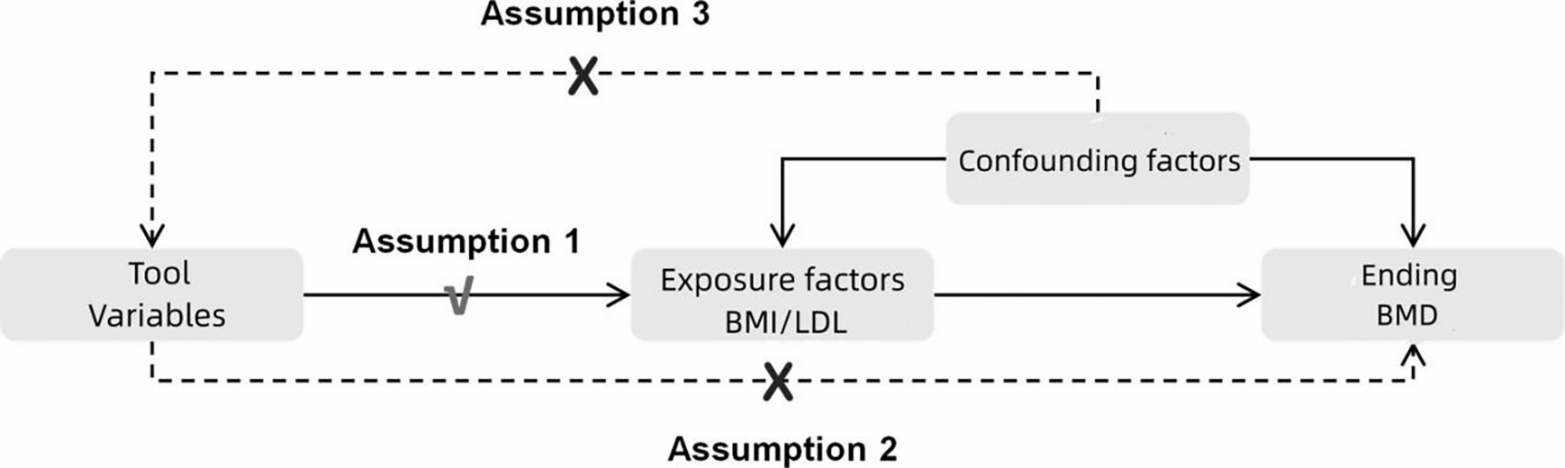
Schematic diagram of the two-sample Mendelian randomization model. **Notes:** MR analysis must adhere to three conditional assumptions [15]: (i) a robust correlation exists between instrumental variables (IVs) and exposure factors; (ii) there is an absence of confounding factors in the correlation between exposure and outcome, specifically, the absence of genetic polymorphisms; (iii) there is no direct effect of IVs on the outcome, with the outcome solely influenced by exposure factors.

**Fig 2:**
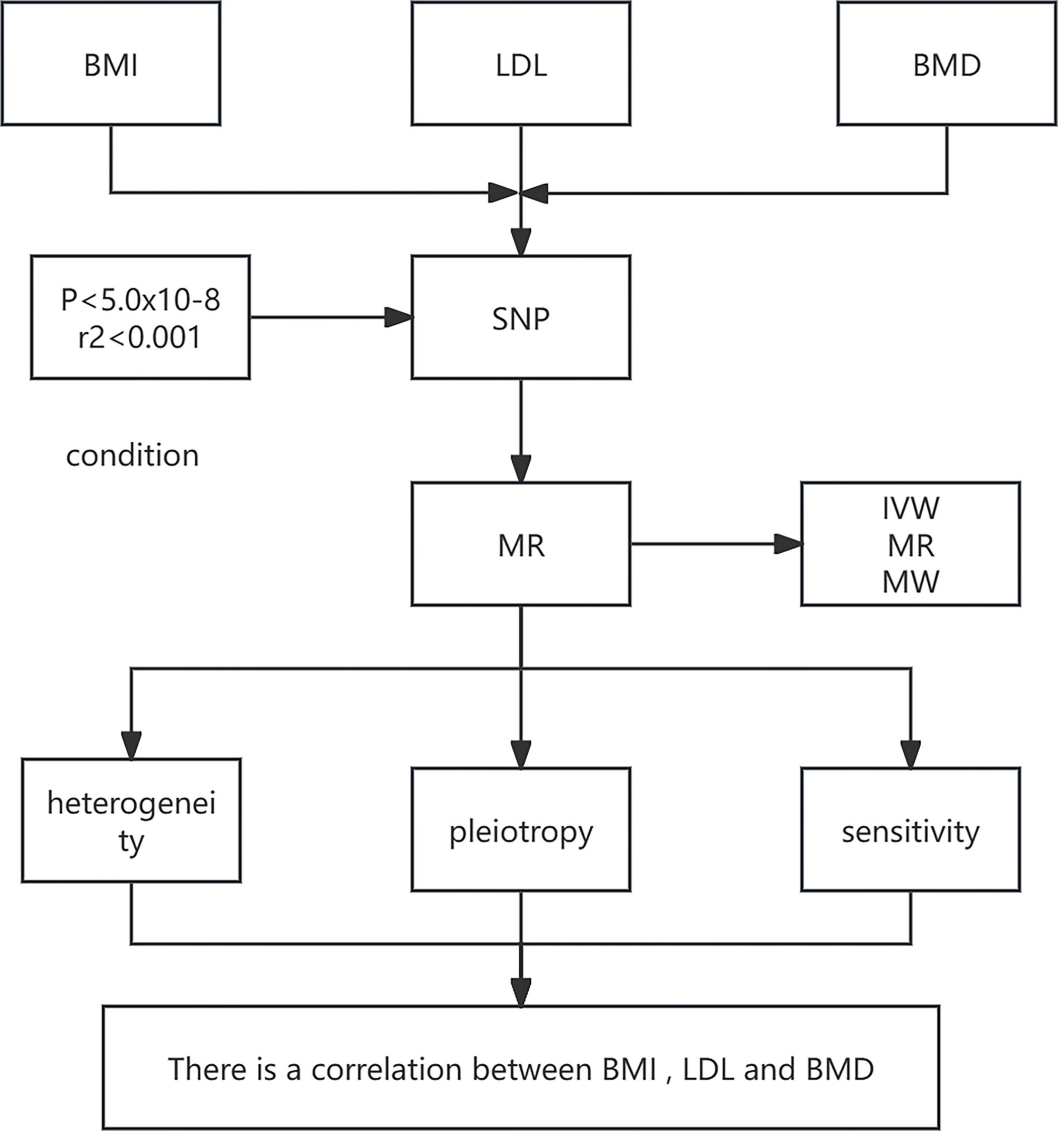
Stepwise execution of Mendelian randomization analysis: Methodological workflow diagram.

The current investigation selected body mass index (BMI) as the exposure factor and procured instrumental variables (IVs) from the exposure dataset in the form of single nucleotide polymorphisms (SNPs) that manifested significant associations with BMI/LDL. The outcome variable of interest was BMD. MR analysis was conducted to investigate the causal association between the exposure and outcome variables. To assess heterogeneity and address potential issues of horizontal pleiotropy, Cochran’s Q test and MR Egger’s test were utilized. Furthermore, sensitivity analysis was carried out to ascertain the robustness and reliability of the derived results [13]. The reliability of MR analysis is predicated on the fulfillment of three conditional assumptions [14]: (i) There exists a robust correlation between instrumental variables and exposure factors. (ii) No confounding factors are present that might influence the relationship between exposure and outcome variables, specifically, no genetic polymorphisms. (iii) Instrumental variables do not assert a direct effect on the outcome, but exclusively influence the outcome via the exposure factors. Refer to **Figs 1** and **2** for a visual depiction of this relationship.

### 2.2 Data sources

Data for Genome-Wide Association Studies (GWAS) on BMI and BMD were independently obtained from the IEU GWAS database (https://gwas.mrcieu.ac.uk/terms/), hosted by the University of Bristol, UK. The BMI dataset comprised 681,275 samples and included 2,336,260 Single Nucleotide Polymorphisms (SNPs). Summary statistics for BMI were sourced from the GIANT consortium. The GIANT Alliance is an international collaboration of researchers from various groups, institutions, countries, and research organizations. The consortium’s primary goal is to identify genetic loci regulating human size and shape, including obesity-related traits such as height, BMI, waist circumference, etc., through meta-analysis of genome-wide association data and other large-scale genetic datasets [16]. Genetic associations with LDL (N = 440,546) were obtained from the UK Biobank [17]. The average age of these participants was 56.9 (range: 39–73) years old, with 54.2% being women. The mean values of lipid concentrations (standard deviation) were as follows: HDL = 1.45 (0.38) mmol/L, LDL = 3.57 (0.87) mmol/L, and TG (median) = 1.50 (1.11) mmol/L. Publicly accessible data for genetic variants associated with Total Body Bone Mineral Density (TB-BMD) were retrieved from the Genetic Factors for Osteoporosis (GEFOS) consortium (http://www.gefos.org/). Summary data from a previous meta-analysis of 30 GWASs on TB-BMD, including 66,628 individuals (56,284 of European ancestry), were utilized to investigate genetic determinants of TB-BMD variation. The TB-BMD SNPs had already been adjusted for covariates (age, weight, height, etc.) via linear regression models [18]. Detailed information is provided in **Table 1**.

**Table 1:** Summary information on the GWAS data from the MR study.

Variables	Trait	Sample size	Number of SNPs	Population	Year
BMI	body mass index	681,275	2,336,260	European	2018
LDL	LDL cholesterol	440546	12321875	European	2020
BMD	Total body bone mineral density	56284	16,162,733	European	2018

### 2.3 Instrumental variables selection

Initially, SNPs demonstrating significant associations with BMI/LDL (selection criteria: P<5.0×10^−8^) were chosen based on the 1,000 whole-genome European population. The linkage disequilibrium parameter (r^2^) was set at 0.001, and the genetic distance was established at 10,000 kb to ensure the independence of each instrumental variable (IV), excluding interference from other IVs. SNPs with strong linkage disequilibrium (r^2^ > 0.8) were subsequently employed to compensate for missing SNPs in the outcome dataset. Finally, exposure and outcome data were extracted from both BMI and BMD datasets, merged, and combined while preserving the corresponding values for exposure and outcome associated with the same effect alleles [19]. The residual SNPs constitute the ultimate instrumental variables related to the exposure. To consider the proportion of SNP phenotypic variation integrated into the Mendelian randomization process, R^2^ values were calculated using the formula R^2^ = 2 ×β^2^ × EAF × (1-EAF)/[2 × β^2^ × EAF × (1-EAF)+ SE^2^ × 2 × N × EAF × (1-EAF)], where β represents the effect estimate of the genetic variant in the exposure GWAS, EAF is the Allele 1 frequency, SE is the standard error, and N is the sample size [20, 21]. Subsequently, the instrumental strength of our SNPs for each socioeconomic trait was assessed using the F-statistic, calculated as F = [(N—k—1)/k] × [R^2^/ (1—R^2^)], where N is the sample size, k is the total number of SNPs in the MR analysis, and R^2^ is the total proportion of phenotypic variation explained by all the SNPs in the MR analysis. An F-statistic greater than 10 indicates that the combined SNPs serve as sufficiently strong instruments to elucidate phenotypic variation, while an F-statistic of 10 or less suggests a weak instrument [21].

### 2.4 Mendelian randomisation

MR analysis is a powerful tool in epidemiological studies. This study began with a two-sample Mendelian randomisation using BMI and LDL as exposures and BMD as an outcome indicator, respectively. As BMI and LDL are correlated in clinical studies, multivariate Mendelian randomisation was performed in order to correct for the results. The main methods used in Mendelian randomisation were inverse variance weighted (IVW), MR-Egger regression and weighted median estimator (WME) for MR analysis. The IVW principle is to weight the inverse of the variance of each instrumental variable as a weight while ensuring that all instrumental variables are valid. The regression does not take into account the intercept term and the final result is a weighted average of the effect values of all instrumental variables [18]. The MR-Egger method differs from IVW in that it takes into account the presence of an intercept term in the regression, and it also uses the inverse of the variance of the outcome as the weight for fit [22]. WME is defined as the median of the weighted empirical density function of the ratio estimates and allows for consistent estimation of causality if at least half of the valid instruments are present in the analysis [23]. The IVW approach provides accurate estimates when all included SNPs meet the criteria for a valid instrumental variable [24]. Therefore, when there were no weak IVs, we used IVW as the primary outcome. However, the weighted median method may provide a reasonable estimate of causality if less than 50% of the weight in the analysis is accounted for by valid instrumental variables [25].

### 2.5 Mediated Mendelian randomisation

**Fig 3:**
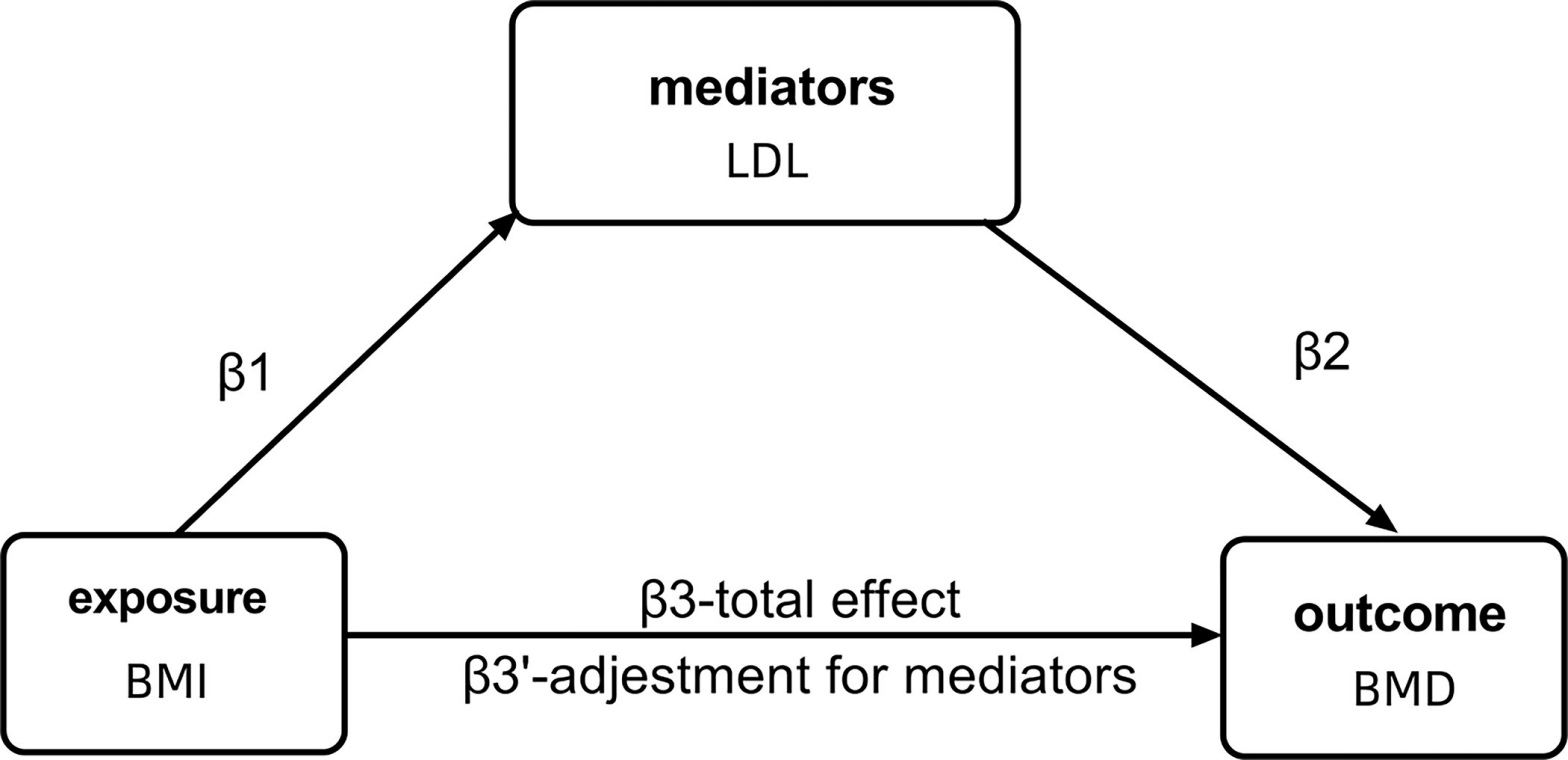
Graph of the proposed mediation by mediators for the association of BMI with BMD. **Notes:** β1 represents the regression coefficients for the association between BMI and mediators, β2 represents the regression coefficients for the association between LDL and BMD, and β3 represents the total effect between BMI and BMD without adjustment for LDL. Additionally, β3’ represents the direct effect between BMI and BMD, considering adjustment for LDL.

To explore the potential mediating role of LDL in the relationship between BMI and BMD, we used a two-step MR approach, as shown in **Fig 3**. The two-step approach is considered less prone to the biases inherent in common multivariate methods [26]. In MVMR, the total effect of each exposure is decomposed into a direct effect and an indirect effect. A mediator was considered to exist if the following conditions were met:1) there was a correlation between BMI and the mediator (β 1); 2) BMI was correlated with BMD but not adjusted for the mediator (β 3); and 3) the mediator was correlated with BMD (β 2). The mediation ratio was calculated as (β 1 x β 2)/(β 3), with β 1 x β 2 being the indirect effect and the total effect being β 3 + β 1 x β2.

### 2.6 Sensitivity analysis

This study implemented a ’leave-one-out’ sensitivity analysis, whereby individual SNPs were eliminated one at a time, to evaluate whether the observed variation was driving the association between the exposure and outcome variables. Secondly, to ascertain the presence of horizontal pleiotropy in this MR analysis, the MR-Egger intercept test was conducted. If the intercept term in the MR-Egger intercept analysis proved statistically significant, it indicated substantial horizontal pleiotropy within the study [27]. Finally, Cochran’s Q statistic was employed in this study to identify heterogeneity. A statistically significant result from the Cochran’s Q statistic test signified a significant level of heterogeneity within the analysis [28].

### 2.7 Statistical analysis

In the present study, the "TwoSampleMR"[29] and "Mendelian randomisation" [30] packages, accessible within RStudio software, were utilised to carry out the exposure and outcome analyses. The outcomes from the Mendelian randomization (MR) analysis were presented as beta (β) values, denoting the impact of BMI and LDL on BMD. Furthermore, the associated 95% confidence intervals (CI) were documented for all causal estimates. A significance threshold of p < 0.05 was implemented to discern statistical significance.

## 3 Results

### 3.1 Two-sample Mendelian randomisation

In the scenario where BMI served as the exposure and BMD as the outcome, a two-sample Mendelian randomization was executed. The IVW results indicated that BMI may function as a protective factor for BMD (β = 0.05, 95% CI 1.01 to 1.09, *P* = 0.01). The MR-Egger regression also yielded congruent results (β = 0.15, 95% CI 1.02 to 1.29, *P* = 0.01). However, the weighted median results did not reveal a statistically significant relationship between BMI and BMD (β = 0.04, 95% CI 0.99 to 1.09, *P* = 0.09). The results from Cochran’s Q test were as follows: IVW analysis (Q = 1701.8, *P* = 1.758784e-45); MR-Egger analysis (Q = 1696.7, *P* = 1.649284e-45). The MR-Egger regression results did not demonstrate a directional pleiotropy effect amongst all genetic variants (intercept, -0.001; 0.09). The funnel plot results showed a point-symmetric distribution, suggesting causal association effects when specific SNPs were used as instrumental variables (IVs). This indicated that causal associations were less susceptible to potential bias (see S1 File). The leave-one-out sensitivity analysis showed that the association between BMI and BMD was not significantly driven by any single SNP (see Fig in the S1 File). Based on these findings, the results in this analysis were not horizontally pleiotropic, suggesting that the IVW analysis results could be adopted as the primary determinant for causality. Hence, it can be concluded that BMI may be a protective factor for BMD.

**Fig 4:**
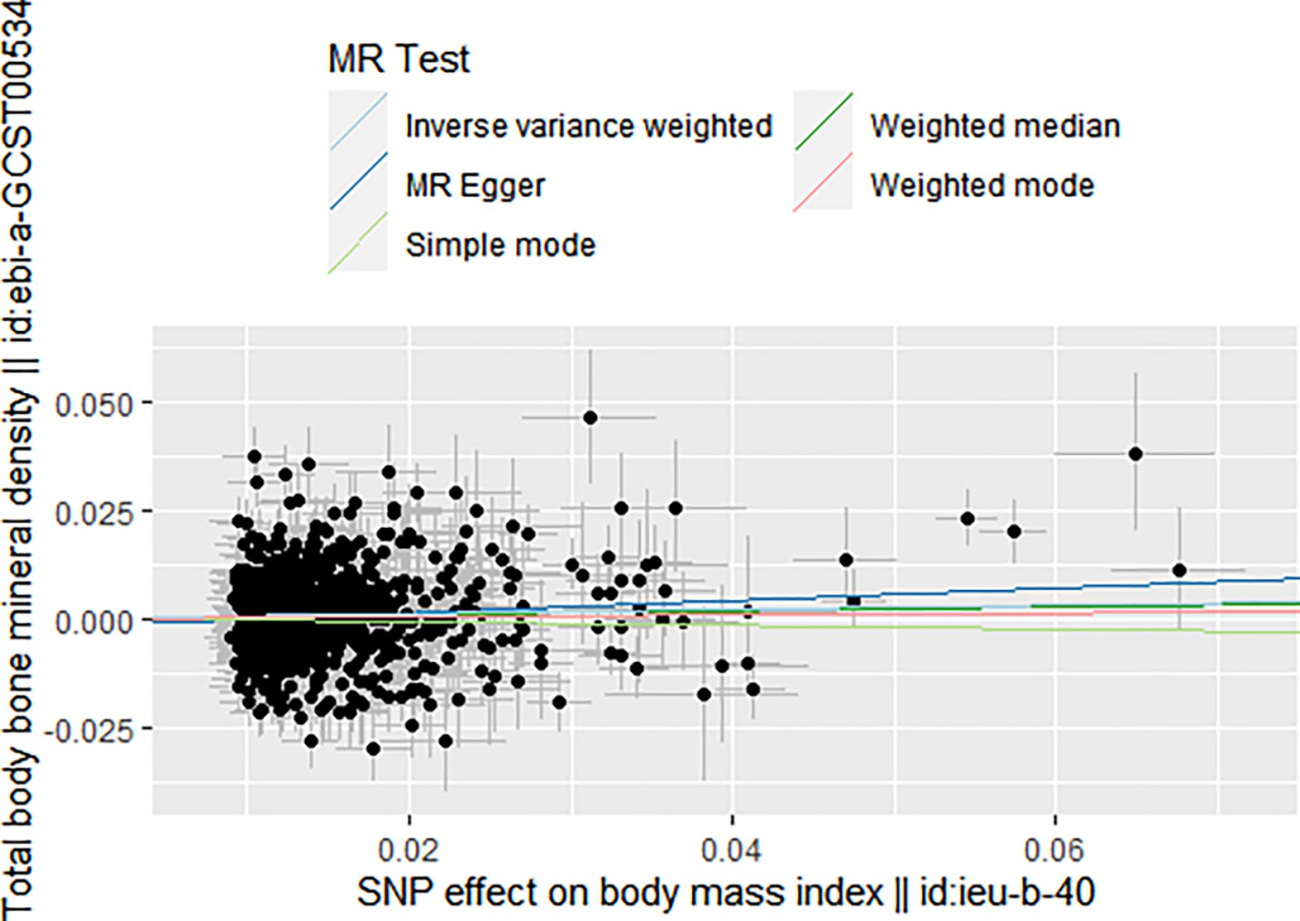
Scatter plot to visualize the causal effect of BMI on BMD.

**Fig 5:**
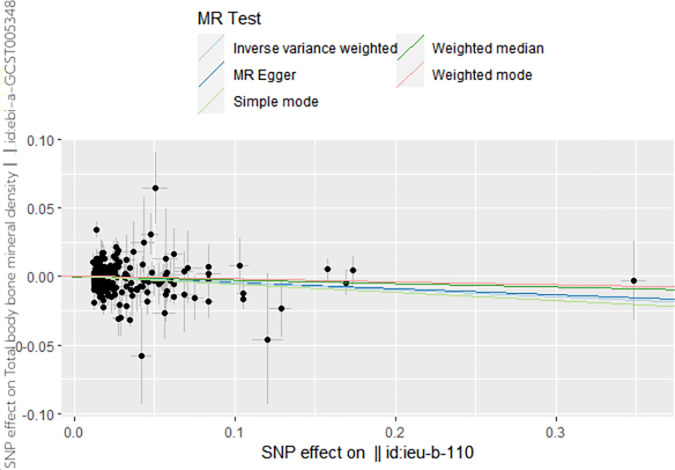
Scatter plot to visualize the causal effect of LDL on BMD.

A two-sample Mendelian randomization was also executed with LDL as the exposure and BMD as the outcome. The IVW results indicated that LDL could be a risk factor for BMD (β = -0.04, 95% CI 0.92 to 0.99, *P* = 0.04). However, MR-Egger regression (β = -0.04, 95% CI 0.9 to 1.02, *P* = 0.25) and weighted median (β = -0.05, 95% CI 0.9 to 1, *P* = 0.09) results did not support a causal association between LDL and BMD. The results of Cochran’s Q test were as follows: IVW analysis (Q = 535.97, *P* = 4.534505e-16); MR-Egger analysis (Q = 535.92, *P* = 3.536905e-31). The MR-Egger regression results did not show any directional pleiotropy effects amongst all genetic variants (intercept, -0.001; 0.86). The funnel plot results illustrated a point-symmetric distribution, suggesting causal association effects when specific SNPs were used as IVs. This indicated that causal associations were less susceptible to potential bias (see S1 File). The leave-one-out sensitivity analysis showed that the association between LDL and BMD was not substantially driven by any single SNP (see Fig in S1 File). The results in this analysis were not horizontally pleiotropic, suggesting that the IVW analysis results could be adopted as the primary determinant for causality. Therefore, it can be inferred that LDL may be a risk factor for BMD. The results are shown in **Table 2**, **Figs 4** and **5.**

**Table 2:** Two-sample MR analysis results under different methods.

Exposure	Outcome	Method	β	95% Cl	*P* value
BMI	BMD	IVW(random effects)	0.05	1.01~1.09	0.01
		Weighted median	0.04	0.99~1.09	0.09
		MR-Egger	0.15	1.02~1.29	0.01
LDL	BMD	IVW(random effects)	-0.04	0.92~0.99	0.04
		Weighted median	-0.04	0.9~1.02	0.25
		MR-Egger	-0.05	0.9–1	0.09

### 3.2 Multivariate Mendelian randomisation

To adjust for potential pleiotropic pathways that might confound the relationship between BMI and LDL, an MVMR model was utilized. In this model, the combined effect of BMI and LDL was used as an exposure for BMD outcomes. The results of the IVW analysis revealed that the previously significant correlation between BMI and BMD, as genetically predicted by UNMR, was attenuated in the MVMR model and was no longer statistically significant (IVW:β = 0.04, 95% CI 0.99 to 1.1, *P = 0*.*07*). Similar results were obtained with the MR-Egger regression (β = 0.04, 95% CI 0.99 to 1.1, *P* = 0.54). In contrast, the negative effect of LDL on BMD remained significant even after adjusting for BMI (IVW: β = -0.08, 95% CI 0.85 to 0.97, *P* = 0.006; MR-Egger: β = -0.08, 95% CI 0.87 to 0.95, *P* = 0.01). The F-statistics in the MVMR model were all greater than 10, indicating that the likelihood of bias in the results is low, enhancing the reliability and stability of the study results. Detailed results are presented in **Table 3.**

**Table 3:** Multivariate MR analysis results under different methods.

Exposure	Outcome	Method	β	95% Cl	*P* value	F-statistics	R^2^
BMI		IVW	0.04	0.99–1.1	0.07	67.17	
		MR-Egger	0.04	0.99–1.1	0.54		
LDL		IVW	-0.08	0.85–0.97	0.006	71.73	
		MR-Egger	-0.08	0.87–0.95	0.01		

### 3.3 Mediated Mendelian randomisation

In order to investigate the potential mediating role of LDL in the relationship between BMI and BMD, a mediated MRanalysis was conducted. The results of this study are detailed in **Table 4**. Given the absence of a direct effect between BMI and BMD, the mediating effect was calculated as an indirect effect (β = β1 x β2). Our study revealed that LDL serves as a mediator in the relationship between BMI and BMD, with the mediating effect estimated to be (β = 0.05, 95% CI 1.04 to 1.05, *P* = 0.04).

**Table 4:** Results of intermediate Mendelian randomisation analysis.

Exposure	Mediated	Outcome	beta	95% CI	P value
BMI		BMD	0.04	-0.01–0.09	0.11
LDL		BMD	-0.07	-0.11–0.02	0.004
BMI		LDL	-0.08	-0.12–0.03	0.0003
BMI	LDL	BMD	0.05	1.04–1.05	0.04

## 4. Discussion

Osteoporosis, cardiovascular disease, and obesity pose significant challenges to public health. The relationship between BMI and BMD remains a subject of debate, with the mechanism of this association not fully understood. In this study, we utilized large-scale GWAS pooled data and employed two-sample MR to examine the causal relationship between BMI and BMD, as well as between LDL and BMD. Given the absence of evidence for horizontal pleiotropy in the study results, we consider the IVW results as the primary determinant for the causal association. Our findings indicate that BMI may potentially delay the progression of osteoporosis, while LDL may pose a risk to BMD. The association between BMI and BMD was observed to be mediated by LDL, suggesting an interconnectedness between osteoporosis and cardiovascular disease. LDL emerges as a risk factor for BMD, underscoring the potential of controlling abnormal lipid levels to mitigate the risk of osteoporosis.

Prior Mendelian randomization investigations have also explored the association between BMI and BMD. For instance, the study conducted by Jidong Song et al. [31] investigated the impact of BMI on BMD at various skeletal sites. Both Song et al. and Soo Ji Lee et al. [32] reported consistent findings with ours, indicating a protective role of BMI against BMD reduction. Nevertheless, no Mendelian randomization study to date has delved into the potential mechanism underlying this relationship. Previous meta-analyses [33] have indicated that excess adipose tissue is associated with dyslipidemia, and obesity serves as a significant risk factor for LDL, with levels of LDL-related markers decreasing as BMI decreases. However, the relationship between LDL and BMD remains contentious, as various studies present conflicting results. For instance, Ersoy et al. [34] demonstrated a positive association between LDL-C and BMD, a Chinese cross-sectional study [35] found no correlation between LDL and BMD, and a Korean study [36] reported a negative association between LDL and BMD. These disparate outcomes may be attributed to differences in study populations, ethnicity, and sample sizes. Our findings align with preceding Mendelian randomization studies [37], suggesting a negative association between LDL and BMD. Supporting evidence for our study includes research indicating that oxidized LDL promotes osteoclast differentiation by inducing osteoclast-associated receptors in endothelial cells [38], and a negative correlation between LDL and BMD was also confirmed by the unidirectional effect between statins that lower LDL-c and BMD [39].

The association between BMI and osteoporosis is indeed a subject of clinical discourse. Some studies propose a positive correlation between BMI levels and BMD, indicating that higher BMI is linked to a reduced risk of osteoporosis [40]. A cross-sectional study conducted in Brazil also implies that increased weight may confer some degree of protection against osteoporosis. Regarding the specific mechanisms underpinning this association, certain researchers hypothesize that it may be ascribed to the heightened mechanical loads experienced by individuals with higher BMI. These mechanical stresses could stimulate osteoblast differentiation and foster bone mineralization, thereby enhancing BMD and decelerating the rate of bone loss in humans [41]. Recent investigations, however, have disclosed that the impact of fat on bone health is not entirely positive. While there exists a threshold below which BMI and BMD exhibit a positive correlation, partially impeding the progression of osteoporosis, above this threshold where the correlation turns negative, obesity is associated with an escalated rate of bone loss and osteoporosis progression [42]. Additionally, a meta-analysis identified a positive correlation between BMD and BMI in cases of moderate obesity (BMI 18.0 to 31.2 kg/m^2^) but a negative correlation in severe obesity (BMI 31.3 to 40.6 kg/m^2^) [43]. These findings align with the outcomes of our study, indicating a causal relationship between BMI and BMD, wherein obesity contributes to an accelerated rate of bone resorption. In contrast to previous observational studies, the current study, employing Mendelian randomization, suggests that the causal connection between BMI and BMD arises from intrinsic genetic factors. This perspective provides specific advantages in comprehending the BMI-BMD relationship, particularly in terms of potential intervention strategies and therapeutic approaches.

The interplay between BMI and BMD is intricate and multifaceted, as indicated by prior research. Modest increases in fat mass could potentially offer both mechanical and biological stimulation to bones. External stimuli can deform and extrude bone cells, leading to an accumulation of these cells. This process might accelerate the rate of bone transformation and the accumulation of bone minerals in the human body, ultimately resulting in increased bone density. Experimental studies have demonstrated that moderate obesity in humans can elevate body fat ratios, effectively preserving bone mass and reducing the rate of bone loss [44]. Simultaneously, moderate obesity may boost the body’s metabolism and stimulate the release of hormones or cytokines beneficial for bone density [45]. Research suggests that moderate obesity can raise the body’s levels of vitamin D, a crucial hormone for regulating bone metabolism. Vitamin D influences the metabolism of calcium and phosphorus in the human intestine, kidney, and bone, while also regulating bone resorption and formation [46]. Studies have shown that obese individuals tend to have higher vitamin D levels than those with a healthy weight, and these elevated vitamin D levels can support bone structure maintenance and promote bone remodeling [47]. Certain research has also found that the quantity of adipocytes and fat accumulation can stimulate leptin secretion. Mild obesity encourages leptin secretion, which, in turn, accelerates the differentiation of mesenchymal stem cells into osteogenic cells, leading to a significant increase in bone mass [48]. In conclusion, the relationship between BMI and BMD is substantial, and BMI may serve as a protective factor for BMD.

In conclusion, the two-sample, two-step Mendelian randomization (MR) employed in this investigation aimed to ascertain a potential causal association between BMI and BMD, as well as between LDL and BMD, revealing a discernible causal relationship among these variables. Consequently, obesity may be considered a protective factor for BMD, while LDL emerged as a risk factor. The findings additionally imply that the interrelation between BMI and BMD is influenced by LDL levels. Thus, interventions targeting the regulation of LDL levels could potentially mitigate the risk of osteoporosis. However, it is essential to acknowledge certain limitations in our study. Firstly, the presence of repeated individuals across various large datasets, from which the SNPs were selected, introduces the possibility of sample overlap between exposure factors and outcome variables, potentially introducing bias. Secondly, the association between BMI and BMD may not adhere to a simple linear relationship but might exhibit a U-shaped correlation, where both high and low BMI levels pose risks. This non-linear relationship might not be effectively captured using Mendelian randomization design. Lastly, the utilization of pooled data from genome-wide association studies lacked individual-specific information, precluding subgroup analyses for variables such as age, sex, disease duration, treatment, and disease typing. This limitation hinders our ability to compare potential differences in causal effects among these subgroups.

In summary, this investigation systematically examined the interplay among BMI, LDL, and bone mineral density utilizing Mendelian randomization methodology. Findings indicate that BMI confers a protective influence on bone mineral density, whereas LDL emerges as a detrimental factor. These results advocate for the consideration of lipid-lowering pharmaceutical interventions to mitigate bone loss, particularly pertinent for individuals grappling with obesity.

## Supporting information

S1 File (ZIP)
