# Supplementary figures and images for: Causal relationships between body mass index, low-density lipoprotein and bone mineral density: Univariable and multivariable Mendelian randomization
Source: PLoS One. 2024 Jun 13;19(6):e0298610. doi: 10.1371/journal.pone.0298610 (PMC11175445; doi:10.1371/journal.pone.0298610)

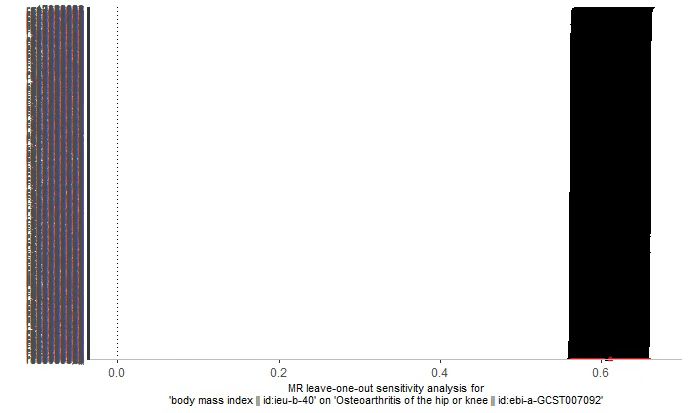

Supplement: S1 File — (ZIP) [file pone.0298610.s001.zip › Supplementary 2 .jpg]

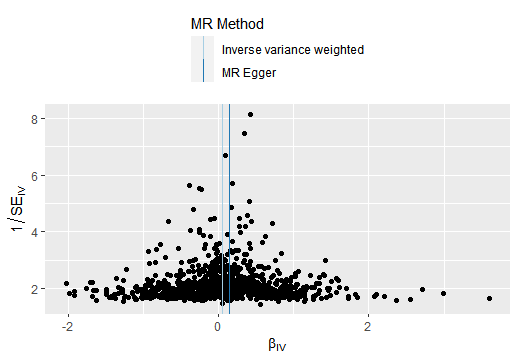

Supplement: S1 File — (ZIP) [file pone.0298610.s001.zip › Supplementary 3 .png]

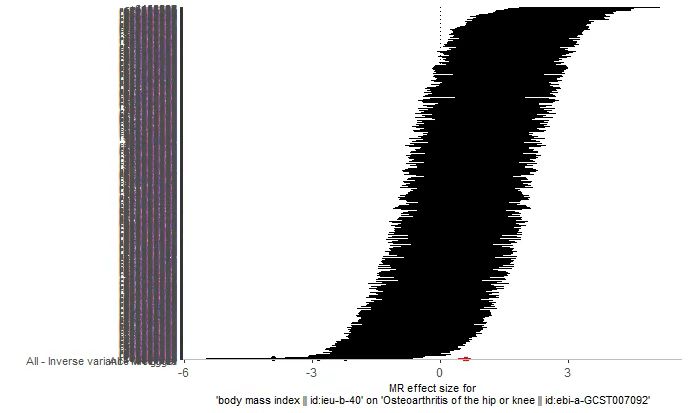

Supplement: S1 File — (ZIP) [file pone.0298610.s001.zip › Supplementary 4 .jpg]

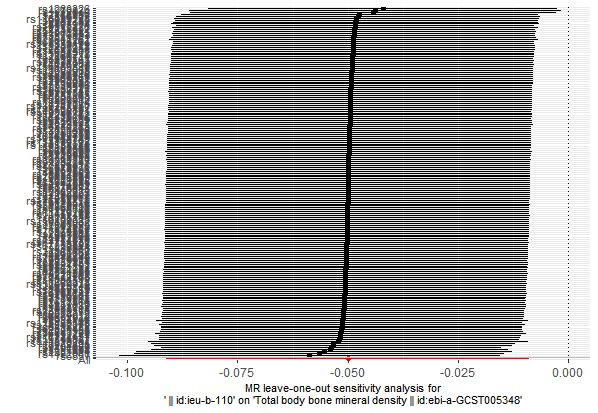

Supplement: S1 File — (ZIP) [file pone.0298610.s001.zip › Supplementary 5 .png]

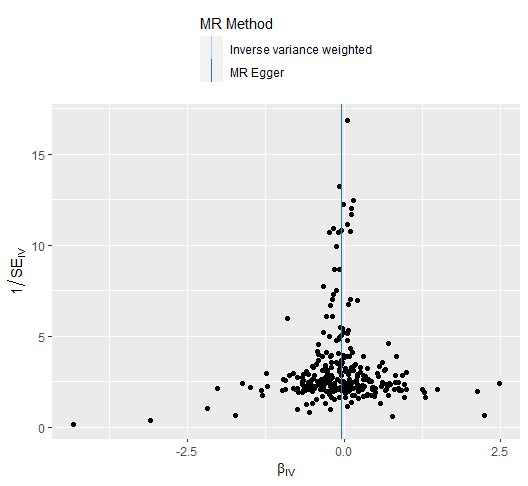

Supplement: S1 File — (ZIP) [file pone.0298610.s001.zip › Supplementary 6 .tiff]

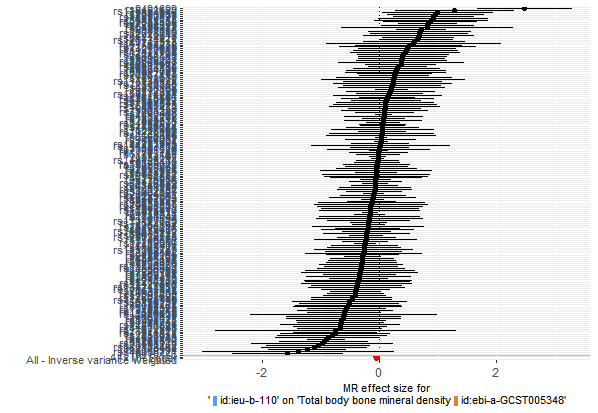

Supplement: S1 File — (ZIP) [file pone.0298610.s001.zip › Supplementary 7 .png]
